# Multilevel analysis of women’s education in Ethiopia

**DOI:** 10.1186/s12905-023-02380-6

**Published:** 2023-04-27

**Authors:** Nuru Mohammed Hussen, Demeke Lakew Workie

**Affiliations:** 1 Statistics Department, Samara University, Semera, Ethiopia; 2 Statistics Department, Bahir Dar University, Bahir Dar, Ethiopia

**Keywords:** Education, Ethiopia, Ethiopia demographic and health survey, Hierarchical data, Multi-level analysis, Single- level approach

## Abstract

**Background:**

Women’s education is the base for faster economic growth, longer life expectancy, lower population growth, improved quality of life, and a high rate of investment return in developing countries. Historically, girls were denied opportunities for schooling in most of the regions and societies of Ethiopia. So this study targeted a multilevel analysis of women’s education in Ethiopia using the 2016 Ethiopian Demographic and Health Survey data.

**Methods:**

Secondary data on women’s data sets were obtained from the 2016 Ethiopia Demographic and Health Survey. A population-based cross-sectional study design was used for the survey. The sampling technique used for the survey was the two-stage sampling technique, which is stratified in the first stage and equal probability systematic selection technique in the second stage. The multi-level ordinal logistic regression model was fitted to identify the determinants of women’s education in Ethiopia.

**Results:**

Among the random sample of 17137 women, the majority, 65.6 percent were rural residents. Somali regional state (75.3 percent) and the capital city Addis Ababa (8.6 percent) had the highest and lowest percentages of women illiteracy respectively than the remaining administrative units of Ethiopia. The minimum values for the fit statistics and the indicative value of the intra-class correlation (68.3%) of the multilevel model showed its appropriateness to the data. Among the predictors in the final multilevel ordinal logistic regression analysis, women’s age at first marriage, residence, and family’s wealth index were significant predictors of women’s education in Ethiopia. Moreover, the estimates from the random effect result revealed that there is more variation in women’s education between the enumeration areas than within the enumeration areas.

**Conclusion:**

A multi-level ordinal logistic regression analysis has determined higher-level differences in women's education that could not be addressed by a single-level approach. So, the application of standard models by ignoring this variation ought to embrace spurious results, then for such hierarchical data, multilevel modeling is recommended.

## Introduction

Education is widely used as an indicator of the status of women and in recent literature as an agent to empower women by widening their knowledge and skills [1]. The birth of endogenous growth theory in the nineteen eighties and also the systematization of human capital augmented Solow- Swan model [2]. This resulted in the venue for enforcing education-centered human capital in cross-country and country-specific growth studies. However, in the nineteen nineties the trend of analyzing the impact of gender-separate education and gender inequality in education on the gross domestic product (GDP) per capita and its growth conjointly emerged in the area of education. Despite the impact of the gender gap, countries with higher levels of women's education expertise have faster economic growth, longer life expectancy, lower population growth, and improved quality of life, and investments in girls' education have a particularly high rate of return in developing countries [3]. Investment in women's education incorporates a higher effect than the other investment in physical or human capital as a result of educated women confer sizeable and tangible advantages on their families and neighborhoods. One more year of a girl’s education will increase her subsequent income; it conjointly reduces fertility, cuts maternal mortality, and improves the health of children. Generally, enhancing women's contribution to development is both an economic and social issue [4].

The issues of gender inequality in Schools within developing countries have been gaining increased attention globally over the last three decades, especially since the 1990 World Conference on ‘Education for All’ in Jomtien, Thailand [5]. This ended in an excess of educational data, particularizing the marginalized position of girls and young women in sub-Saharan Africa. However, the information about the life experiences of female students who have succeeded within harsh educational systems despite highly unfavorable odds was too little. Despite efforts to reach gender equality in enrolment, the United Nations Educational, Scientific and Cultural Organization (UNESCO, 2000) narrates that, between 1990 and 1998, the gender gap, as measured by the gross enrolment ratio, has grown in Sub-Saharan African countries such as Ethiopia. In addition, as students move up the educational ladder the gender gap between the number of males and females significantly rises. When this gap is examined in terms of rural and urban inequality, it was founded that girls living in rural communities face a “double disadvantage”, consistent with the United Nations Children’s Fund (UNICEF, 2000). A study conducted by Brock and Cammish (1991) revealed the lowest enrolments of women and therefore the largest gender gaps are inevitably occurred within the poorest and least economically developed areas, especially in the rural communities where educational provision is poor, among children of the poorest families, and among children of ethnic minorities [6].

A brief review of literature on girls’ education in Ethiopia followed background information in sub-Saharan Africa. In traditional Ethiopia, girls have been denied schooling opportunities in most regions and societies. Their denial of education goes back to the old traditional schooling systems. Additionally, the Orthodox Church and Mosques were major institutions responsible for the discrimination against female education [7]. The Government of Ethiopia has been taking several measures to reduce the gender gap in education and admirable trends have been observed in recent years. The Government has also incorporated gender issues as one of the prior agendas in its policies and programs. The Education Sector Development Program III (ESDP III), which continues from ESDP I and II, delineated special steps and measures to reduce gender gaps in enrollment, academic performance, and successful graduation, and it suggests several specific activities at the regional, woreda, and school levels [8]. There are the methods and actions undertaken throughout the ESDP III period of 2005/06 up to 2010/11. Similarly, measures have been embraced in the draft of ESDP IV, which was planned to be enforced over the 2010/11 – 2014/15 period. Despite those actions, geographical location, maternal socioeconomic and demographic characteristics, etc., still pose challenges. As a result, identifying enumeration areas and household-specific determinants of women is critical for planning and implementing interventions and taking action to address the burden of women's illiteracy in Ethiopia. Many qualitative and descriptive studies were conducted at different components of the globe on women's education and management [9–13]. However, in view of the above literature, this study is intended on the multi-level analysis of women's education in Ethiopia by using the 2016 Ethiopia Demographic and Health Survey data.

## Materials and methods

### Data and sampling design

This study uses secondary data set from the 2016 Ethiopia Demographic and Health Survey (EDHS) which was the fourth demographic and health survey. From January 18, 2016, to June 27, 2016, the survey used a population-based cross-sectional study design in which women were interviewed for information on their education history. Each of the interviewed respondents was either a permanent resident of the designated households or a visitor who stayed the night before the survey. EDHS was strengthened by the Central Statistical Agency (CSA) with support obtained from the Ministry of Health. This survey was conducted in Ethiopia as a part of the worldwide DHS project, a USAID-funded project providing support and technical assistance with the enforcement of population and health surveys in countries worldwide.

The sampling frame for EDHS 2016 was the same as for EDHS 2007 (CSA, 2008). The EDHS 2016 uses a census frame of 84,915 enumeration areas (EAs) created for the 2007 population and housing census. An EA is a geographical area with an average of 181 households. Ethiopia has been divided administratively into 9 nation-states and 2 administrative cities. The sample for EDHS 2016 was stratified and selected in two stages. Every region was stratified by dividing it into urban and rural areas except the Addis Ababa region because it is entirely urban. Samples of EAs were selected independently in every stratum in 2 stages. Among the 645 selected EAs in the first stage, 202 were urban and 443 were rural, and in the second stage, a fixed number of 28 households per enumeration areas were chosen using an equal probability systematic selection from the newly created household listing.

### Variables in the study

The response variable of this study was the education level of women. According to [14] education level is ordered as no education, primary education, secondary education, and higher education. However, women’s age at first marriage, frequency of listening to the radio, frequency of reading newspapers and magazines, frequency of watching television, family’s Wealth index, and Religion were potential lower level predictors of women’s education, while childhood place of residence, was the potential higher( enumeration area) level predictor [15]. Secondary data was managed with SPSS-26 and analyzed using R-3.6.3 and SAS- 9.4. The model selection was made between the single level and multilevel approach by using Akaike Information Criteria (AIC) and Bayesian Information Criteria (BIC) because of their non-nested nature.

### Statistical analysis

In this study, a multilevel ordinal logistic regression model was employed to predict the educational level of women as a function of the above-listed predictors. Due to the stratified nature of data in EDHS, women were naturally nested into enumeration areas and enumeration areas are nested into regions. Hence, keeping in view the hierarchically clustered nature of the data, the multilevel regression model was used to avoid possible underestimation of parameters from a single-level model [16]. The benefit of multilevel models is that they properly account for the correlation structure of data, which is common in social sciences and multistage survey sampling. To account for the data's hierarchical nature, we use state of residence as the level-2 variable under which the respondents are nested. Thus, respondent-level variables are the level-1 variables used in this study to predict women’s education level. Our outcome variable has four levels and is ordinal polytomous. To compute the cumulative odds for each response category, the model for such data employs a multinomial distribution and a cumulative logit link function [17]. All discussions of an ordinal response variable impose a restrictive assumption on the data, known as the assumption of proportional or identical odds. This assumption implies that the effect of any explanatory variable remains constant regardless of how the data is split. The assumption of proportional odds implies that the effect of a predictor is assumed to be the same. "Partial" proportional odds models are collections of models in which some predictors have non-proportional odds, but Proportionality tests for single-level approaches have been shown to be statistically ineffective [17, 18]. Fitting the underlying series of hierarchical logistic models and examining deviation from consistent patterns in variable effects among predictors are ad hoc methods for investigating proportionality in the multilevel framework. Although other link options, such as the complimentary log–log link for CR models, are currently available in a few statistical packages, most software for the analysis of multilevel ordinal data will fit the proportional odds model, which is based on the cumulative logit link. As estimation and software methods for ordinal random- or mixed-effects models improve, it is likely that these various alternatives, including those that allow for partial-proportional odds, will become more widely available.

### The hierarchical proportional odds model

The most popular method for analyzing hierarchical ordinal data is the proportional odds model. The cumulative probability of success (using the ascending option) across the K-1 cumulative splits for a K-level ordinal outcome is based on a model using the cumulative logit link for the response, ${R}_{ij}$ for ${i}^{th}$ women in the ${j}^{th}$ enumeration area. Utilizing terminology from [19], the model is characterized by levels as follows:

- Level 1: $${\eta }_{kij}=ln\left(\frac{P\left({R}_{ij}\le K\right)}{P\left({R}_{ij}>K\right)}\right)= {\beta }_{0j}+{\sum }_{q=1}^{Q}{\beta }_{qj}{x}_{qij}+{\sum }_{j=1}^{q}{\beta }_{0j}{Z}_{j}+{\sum }_{k=2}^{k-1}{D}_{kij}{\varepsilon }_{k}$$ and
- Level 2:$${\beta }_{qj}= {\gamma }_{q0} + {\sum }_{s=1}^{{S}_{q}}{\gamma }_{qs}{W}_{sj}+{U}_{qj}$$  

Where ${\eta }_{kij}$ is the logit prediction for ${k}^{th}$ cumulative comparison and for the ${i}^{th}$ women in the ${j}^{th}$ enumeration area. Remember that the logit is the natural log of the success probability odds. To get from logits to odds to the predicted probability of success, ${\pi }_{ij},$ given a vector of predictor variables (where x includes level-one and level-two predictors), we use the relationship:

$${\pi }_{kij } = \frac{exp\left({\eta }_{kij}\right)}{1+exp\left({\eta }_{kij}\right)} = \frac{{odds}_{kij}}{{1 +odds}_{kij}}$$

The model generates a series of K-1 probabilities for each person, each representing the probability of the response being in or below a given category based on the set of predictors. Because all responses must be at or below the ${k}^{th}$ level, the ${k}^{th}$ probability would always equal 1.0. Given the Q women-level predictors, the regression equation at level one provides a unique set of intercept and regression coefficients for each enumeration area. The proportional odds assumption holds that these slopes are constant across all K-1 cumulative splits to the data, though they do vary from group to group. The level two residual terms capture the variability in intercepts and slopes across groups at the group level (level two), ${U}_{qj}$. Variation in the random regression parameter estimates can be modeled using level-two predictors, Wsj that may vary for each regression coefficient from level one. The gamma coefficients at level two are fixed regression coefficients. The residuals at level two decrease as the explanation of the level-one random coefficients improve due to the addition of appropriate level-two predictors. With a variance/covariance matrix, these residuals are assumed to be normally distributed.

## Results

### Percentage distribution of women’s education by regions

According to EDHS 2016 report educational level was classified as no education, primary education, secondary education, and higher education. There were variations between the nine regional states and two administrative cities of Ethiopia based on the educational level of randomly selected women in Ethiopia. Accordingly, the Somali regional state (75.3 percent) and the capital city Addis Ababa (8.6 percent) had the highest and lowest percentages of women illiteracy respectively than the remaining administrative units of Ethiopia. Furthermore, there was a variation in women's enrolment in higher education, with the capital city Addis Ababa (24%) and the Somali regional state (2.0%) having the highest and lowest percentages of enrolment in higher education respectively (Table 1).

**Table 1:** Percentage distribution of women’s education in Ethiopia by regions

Region	No education	Primary	Secondary	Higher
Tigray	43.0	32.2	18.5	6.3
Afar	68.7	24.3	4.1	2.8
Amhara	54.1	28	12.5	5.4
Oromia	51.1	36.8	8.2	3.8
Somali	75.3	18.2	4.5	2.0
Benishangul-Gumuz	46.7	37.5	9.9	5.9
SNNPR	43.9	42.7	9.7	3.7
Gambela	26.7	38.7	22.1	12.5
Harari	36.1	34.5	17.3	12.0
Addis Ababa	8.6	37.2	30.2	24.0
Dire Dawa	33.3	38.2	18.7	9.8

### Women’s education and its socio-demographic features

The majority, 65.6 percent, of the 17133 women in the EDHS 2016 random sample were rural residents, while only 34.4 percent were urban residents. Regarding their religion, 41.7 percent of the sampled women were orthodox, with the remaining 39 percent, 17.4 percent, 0.7 percent, 0.6 percent, and 0.5 percent Muslim, protestant, traditional, catholic, and other religions respectively. Furthermore, the majority of them were within the poorest families (26.6%), followed by 16.6%, 17.9%, 17.6%, and 21.3 percent within the middle, richer, and richest families, respectively. Their average age at the time of their first marriage was 23.3 years. Furthermore, a statistically significant chi-square statistic indicates a significant relationship between women's education and the study's corresponding predictors (Table 2).

**Table 2:** Descriptive statistics

Variables	Categories	Percent	*X*^2^- value(*P*-value)
Place of Residence	Urban	34.4	4431.346(< .0001)
	Rural	65.6	
Religion	Orthodox	41.7	28.03(0.021)
	Catholic	0.6	
	Protestant	17.4	
	Muslim	39	
	Traditional	0.7	
	Others	0.5	
Wealth index	Poorest	26.6	2869.62(< .0001)
	Poorer	16.6	
	Middle	17.9	
	Richer	17.6	
	Richest	21.3	
Listening radio	Not at all	44.2	4.138(0.638)
	Less than once a week	23.6	
	At least once a week	32.2	
Reading newspapers and magazines	Not at all	69.4	6.477(0.378)
	Less than once a week	20.3	
	At least once a week	10.3	
Watching Television	Not at all	48.8	5.769(0.450)
	Less than once a week	24.3	
	At least once a week	26.9	

### Model selection

It is necessary to compare different alternative models in order to select the best and final model that best fits the data. In order to do so, it is important to use three statistics; Akaike information criteria (AIC), Bayesian information criteria, and the likelihood ratio test [20]. Among the series of models, the best one is the one with the smallest value of AIC and BIC for non-nested models, and if there exist nested models, the selection will be made by the significance of the likelihood ratio test. In this study, we have two non-nested models; single-level and multi-level models. As a result of the minimum values for the fit statistics and the indicative value of the intra-class correlation, the multilevel model fits the data better than the single-level model (Table 3).

**Table 3:** Model selection

Fit statistics	Single level model	Multilevel model
AIC	24422.334	20658.016
BIC	24444.002	20723.021
-2 Log L	24416.334	20640.016

When we are interested to analyze on a single level by standard regression models the model fitting is quite easy, but when we have hierarchical data with predictors at different levels, the model fitting process should include a number of steps. While fitting the model with no predictors (i.e. the intercept-only model) is the most important step in multilevel analysis.

$${Y}_{ij}= {\gamma }_{00}+ {u}_{0j}+ {e}_{ij}$$

where:

Y_ij_ represents the education level of the ${i}^{th}$ women in ${j}^{th}$ enumeration area,

γ_00_ represents the overall log odds of education across all women and enumeration areas.

u_0j_ & e_ij_ represent the residual errors at the enumeration areas and women level respectively with their respective variances as ${\upsigma }^{2}{u}_{0}\,and\,{\upsigma }^{2}{\varepsilon }_{0}$ where ${\varepsilon }_{ij}\sim N\left(0,{\sigma }_{\varepsilon }^{2}\right)$.

As a result of the maximum likely hood estimation, the residual error variance at the women and enumeration area levels was estimated to be 0.3013 and 0.6507, respectively. We can conclude that there is more variation among the different enumeration areas (0.6507) than within the enumeration areas (0.3013). This will be discussed further below when we calculate the intra-class correlation ($\rho$), the proportion of variance explained due to the grouping structure.

$$\rho =\frac{{\upsigma }^{2}{u}_{0}}{{\upsigma }^{2}{\varepsilon }_{0}+{\upsigma }^{2} {u}_{0}} = \frac{0.6507}{0.3013+0.6507} = 0.683$$

This result implied that 68.3 percent of the variation in women’s education occurred at the enumeration area level, with the remaining 31.7 percent occurring between women. This is evidence of the need for multilevel analysis than a standard single-level analysis (Table 4).

**Table 4:** Intercept only model

**Estimates for fixed effects**						
Effect	Education	$\widehat{\beta }$( Exp $\left(\widehat{\beta }\right)$)	SE	Pr >|t|	Lower	Upper
Intercept	No	-0.2159(0.8)	0.01537	< .0001	-0.246	-0.186
	Primary	-1.2498(0.29)	0.01836	< .0001	-1.286	-1.214
	Secondary	-2.4519(0.09)	0.02827	< .0001	-2.507	-2.396
**Estimates for random effects**						
Covariance parameter	Estimate	SE				
Intercept(U_oj_)	0.6507	0.09630				
Residual(e_ij_)	0.3013	0.01420				

Key: Exp $\left(\widehat{\beta }\right)$: Crude Odds ratio, *SE* Standard Error

### Variable selection

When modeling with many independent variables, the goal is typically to select those variables which yield the "best" model within the scientific context of the problem. To achieve this "best" model, a basic plan for selecting the variables for the model and assessing the adequacy of the model in terms of the individual variables as well as the overall fit of the model is required. It is also highlighted in that successful modeling of a complex data set is part science, part statistical methods, and part experience and common [21].

### Univariable analysis

In order to determine the importance of each predictor, a purpose full variable selection procedure must begin with a single covariate analysis approach at a 25% level to "screen" out potentially significant variables for consideration in the multivariable model. Based on this analysis, place of residence, religion, age at first marriage, and family’s wealth index were statistically significant at a 25% level and then considered in multivariable analysis (Table 5).

**Table 5:** Univariable analysis

Variable	Num DF	Den DF	*F* Value	Pr > F
Residence	1	17133	3993.81	< .0001
Religion	5	17129	3.27	0.0060
Wealth index	4	17130	651.77	< .0001
Age at marriage	1	17133	4237.52	< .0001
Listening radio	2	17132	0.77	0.4651
Reading newspapers	2	17132	0.01	0.9944
Watching television	2	17132	0.40	0.6722

Key: *Num DF* Degrees of Freedom for the numerator of F distribution, *Den DF* Degrees of Freedom for the denominator of F distribution

### Multivariable analysis

This step consists of all the significant variables at the univariable analysis that have decrement on the -2 Log L statistics than the intercept-only model. Accordingly, results from the final multilevel ordinal logistic regression model revealed that women's age at first marriage, place of residence, and family wealth index were the significant predictors of women’s education in Ethiopia. The random effect estimates revealed that there was more variation between the enumeration areas (0.5253) than within the enumeration areas (0.2166). Furthermore, the variation among rural and urban areas was more significant (0.3834) on the level of women’s education (Table 6).

**Table 6:** Parameter estimates for multi-level ordinal logistic regression model

**Estimates for fixed effects**						
Variable	Categories	$\widehat{{\varvec{\beta}}}$( Exp$\left(\widehat{{\varvec{\beta}}}\right)$)	SE	Pr >|t|	Lower	Upper
Intercept	No education	-0.4241(0.65)	0.0201	< .0001	-0.4635	-0.3845
Intercept	Primary	-1.0542(0.35)	0.0201	< .0001	-1.0936	-1.0148
Intercept	Secondary	-2.4058(0.09)	0.0202	< .0001	-2.4453	-2.3662
Place of residence	Urban	1.9254(6.86)	0.0345	< .0001	1.8578	1.9930
	Rural	0				
Religion	Orthodox	-0.1449(0.86)	0.1996	0.4678	-0.5363	0.2464
	Catholic	-0.4220(0.66)	0.2708	0.1192	-0.9529	0.1089
	Protestant	-0.2279(0.80)	0.2015	0.2581	-0.6228	0.1670
	Muslim	-0.1529(0.86)	0.1997	0.4439	-0.5444	0.2386
	Others	-0.5768(0.56)	0.2601	0.0266	-1.0866	-0.0669
	Traditional	0				
Wealth index	poorest	-0.4883(0.61)	0.0457	< .0001	-0.5779	-0.3987
	Poorer	-0.9213(0.40)	0.0464	< .0001	-1.0122	-0.8304
	middle	-1.2761(0.28)	0.0490	< .0001	-1.3721	-1.1801
	Richer	-1.8117(0.16)	0.0470	< .0001	-1.9038	-1.7196
	Richest	0				
Marriage	In year	0.1272(1.14)	0.0033	< .0001	0.1206	0.1337
**Estimates for Random effects**						
**Covariance parameter**	**Estimate**	**SE**				
Intercept(U_oj_)	0.5253	0.07744				
Residence(U_1j_)	0.3834	0.0215				
Residual(e_ij_)	0.2166	0.01021				

Key: Exp$\left(\widehat{\beta }\right)$: Adjusted Odds ratio

## Discussion

The labeled intercept for no education was -0.4241, which implies that the estimated log odds of falling into the category no education versus all other categories while all the predictors are zero was -0.4241. Thus, the estimated odds of no education across all women and enumeration areas was exp(-0.4241) = 0.65 when all covariates had zero values and all factors were at the reference level. The labeled intercept for primary education was -1.0542, implying that the overall estimated log odds of falling into primary education versus secondary and higher education while all the predictors are zero was -1.0542. Thus, the estimated odds of primary education across all women and enumeration areas was exp(-1.0542) = 0.35 when all covariates had zero values and all factors were at the reference level. The labeled intercept for secondary education was -2.4058, which implies the estimated log odds of falling into secondary education versus higher education while all the predictors are zero was -2.4058. Thus, the estimated odds of secondary education across all women and enumeration areas was exp(-2.4058) = 0.09 when all covariates had zero values and all factors were at the reference level.

The estimated slope of women's age at first marriage was 0.1272, with an odds ratio of exp(0.1272) = 1.14 (*P* = 0.0001), indicating the existence of a positive relation between women’s age at first marriage and their education level. More specifically, as women's age at first marriage increased by one year, the odds of women's education being one level higher increased by about 14.0 percent, while all other variables remained constant. The result was coherent with [22–24].

The estimated slope for the education level of women from urban areas was 1.9254, with the estimated odds ratio of exp(1.9254) = 6.86 (*P* value < 0.0001), indicating that women from rural areas were less likely to get education as compared with urban residents. More specifically, the odds of women’s education level among rural residents was 68.6% less than those of urban residents, keeping all other variables constant. The result was consistent with [25–27], where women from rural areas were less likely to attain secondary and higher education as compared with urban residents.

The estimated slope for the education level of women within the poorest, poorer, middle, and richer families with the corresponding estimated odds ratio was -0.4883 (0.61), -0.9213 (0.40), -1.2761 (0.28), and -1.8117 (0.16), respectively, indicating women from middle- and upper-income families are more likely than those from low-income families to enter school and progress all the way to the university level. More specifically, the odds of women’s education level among women within the poorest, poorer, middle, and richer families were about 61%, 40%, 28%, and 16% respectively less than those among the richest families. The result was consistent with [27–31].

## Conclusions

Unobserved enumeration area level differences in women’s education that cannot be addressed by a single-level approach were determined using a multi-level ordinal logistic regression analysis. The descriptive part of the data revealed regional variations in the distribution of women's education, such as higher and lower percentages of women enrolled in higher education reported from the capital city (Addis Abeba) and Somali regional states, respectively. Inferentially, the multilevel approach was a better fit than the single-level approach due to the minimum value for the fit statistics, and the value of the intra-class correlation was also evidence of its necessity. Among the predictors within the multilevel ordinal logistic regression model, family’s wealth index, childhood place of residence, and age at first marriage were significant predictors for women’s education level in Ethiopia. These findings have valuable policy implications for intervention and program design. Especially for the federal government, the Ministry of Education and Regional Education Bureaus must enforce the legal age of marriage and close monitoring of rural residents is important. This approach incorporates the effect of higher and lower level factors separately and takes into account the effect of the grouping structure in the case of hierarchical data, using single-level analysis by ignoring such grouping structure may provide spurious results, so the multilevel approach is recommended for making effective decisions with such hierarchical data.

## Data Availability

The data set used for this study was accessed from the Measure DHS website (http://www.measuredhs.com).

## Abbreviations

AIC: Akaike Information Criteria
BIC: Bayesian Information Criteria
CSA: Central Statistical Agency
DF: Degree of Freedom
EA: Enumeration Area
EDHS: Ethiopia Demographic and Health Survey
ESDP: Education Sector Development Program
GDP: Gross Domestic Product
LL: Log-Likelihood
UNESCO: United Nations Educational, Scientific and Cultural Organization
UNICEF: United Nations Children’s Fund
USAID: United States Agency for International Development
